# Achieving Excellent Strength-Ductility Balance in Single-Phase CoCrNiV Multi-Principal Element Alloy

**DOI:** 10.3390/ma16196530

**Published:** 2023-10-01

**Authors:** Zengyu Ni, Ziyue Li, Rui Shen, Siyuan Peng, Haile Yan, Yanzhong Tian

**Affiliations:** 1 Key Laboratory for Anisotropy and Texture of Materials (Ministry of Education), School of Materials Science and Engineering, Northeastern University, Shenyang 110819, China; 2 Research Center for Metallic Wires, Northeastern University, Shenyang 110819, China

**Keywords:** CoCrNiV alloy, multi-principal element alloy (MPEA), stacking fault, solid solution strengthening, fine grain (FG)

## Abstract

CoCrNi alloys exhibit excellent strength and ductility. In this work, the CoCrNiV multi-principal alloy with single-phase fine grained (FG) structure was prepared by rolling and heat treatment. The characteristics of deformation microstructures and mechanical properties were systematically investigated by scanning electron microscope (SEM) and transmission electron microscope (TEM). The results indicate that the CoCrNiV alloy successfully attains a yield strength of 1060 MPa while maintaining a uniform elongation of 24.1%. The enhanced strength originates from FG structure and severe lattice distortion induced by V addition. Meanwhile, the exceptional ductility arises from the stable strain-hardening ability facilitated by dislocations and stacking faults. The deformation mechanisms and the optimization strategies for attaining both strength and ductility are thoroughly discussed.

## 1. Introduction

Multi-principal element alloys (MPEA), as a novel type of metallic crystalline materials, have been attracting much attention in the materials research community [1,2,3,4,5,6,7]. The concept of MPEA offers significant compositional variability, enabling the alloy to activate multiple deformation mechanisms to achieve desired properties, such as high hardness [8], high strength [8,9], a remarkable strength-ductility synergy [10], exceptional oxidation resistance [11,12], excellent corrosion resistance [13], superior wear resistance [14], and high radiation resistance [15] and so on. In recent years, due to its advanced alloy design concepts and outstanding properties, MPEAs featuring a single-phase face-centered cubic (FCC) structure have garnered significant research interest. Among the various single-phase FCC MPEAs, the CoCrFeMnNi alloy has been extensively investigated. Compared with room temperature, the CoCrFeMnNi alloy had better strength and ductility at 77 K, which were attributed to the synergistic effect of various deformation mechanisms such as dislocation slip, stacking fault and nanotwin [16]. Furthermore, Laplanche et al. [17] compared the mechanical properties of CoCrNi and CoCrFeMnNi alloys at 293 K and 77 K, respectively. The stacking fault energy (SFE) of CoCrNi was observed to be 25% lower than that of CoCrFeMnNi. And the yield strength and strain-hardening rate of CoCrNi were higher than CoCrFeMnNi, which made CoCrNi alloy reach the critical stress of twinning at lower strain. Consequently, the CoCrNi MPEA exhibited superior mechanical properties compared to the CoCrFeMnNi MPEA.

In order to further improve the yield strength of FCC MPEAs, the researchers increased the lattice friction stress by replacing or adding other alloying elements. The volume mismatch was caused by the difference of atomic size, which led to serious lattice distortion effect and hindered the dislocation movement to strengthen the materials [18,19,20]. For instance, the addition of elements such as C [21,22], Ti [23], Al [23,24,25], Mo [26,27], and others to CoCrNi or CoCrFeMnNi MPEAs can significantly improve the mechanical properties of the alloys. The addition of Al element to the CoCrNi alloy resulted in the formation of (CoCrNi)_87_Al_13_. Compared to the CoCrNi alloy, the (CoCrNi)_87_Al_13_ alloy exhibited an increased yield strength from 346 to 578 MPa and an improved ultimate tensile strength from 605 to 960 MPa, and its ultimate elongation remained at 24% [24]. As a result, exploiting lattice distortion to influence solid solution strengthening has become one of the primary approaches to enhance the mechanical properties of MPEAs.

The V element was considered as the most effective strengthening element for both FCC and BCC MPEAs due to its unique atomic volume, which created a significant mismatch with other primary elements [20,28]. Sohn et al. [29] used V to replace Cr in CrCoNi alloys to obtain a disordered VCoNi alloy, and verified by density functional theory (DFT) simulation that the addition of V increased the fluctuation of atomic spacing and caused severe lattice distortion. Consequently, the VCoNi alloy with FCC structure exhibited a yield strength of 1 GPa, primarily due to the high solid solution strengthening and grain boundary strengthening effects. Chen et al. [30] designed an UFG V_0.5_Cr_0.5_CoNi alloy with a stable dual-phase structure and exceptional overall mechanical properties by incorporating 16.7% V content into the CoCrNi MPEAs. However, both the VCoNi and V_0.5_Cr_0.5_CoNi alloys contained a high content of V. Therefore, it was necessary to explore low V alloys to reduce the manufacturing cost and investigate the microstructure and mechanical properties. In this study, through the preliminary experiment on the process parameters of a wide range of exploration, the cold-rolled samples were annealed at 1023 K and 1073 K for 20 min to obtain a single-phase FCC Co_33_Cr_24_Ni_33_V_10_ alloy with low V content, and the deformation mechanism of high strength and high plasticity was explored by tensile deformation and microstructure characterization. This work introduces a novel strategy for optimizing comprehensive mechanical properties by designing recrystallized fine grains and is expected to be used to explore the excellent mechanical properties of MPEAs in different systems.

## 2. Materials and Methods

**Figure 1:**
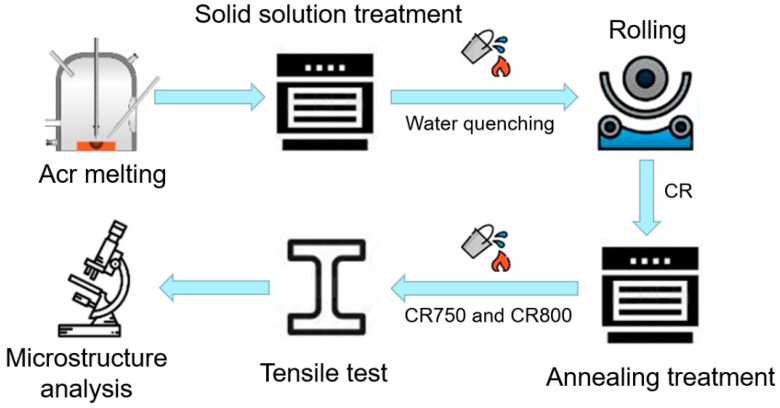
The flowchart of experimental methods.

Co_33_Cr_24_Ni_33_V_10_ alloy was prepared using raw materials with purity of 99.99 wt.%. The composition of the studied alloy was 32.58Co-24.66Cr-32.29Ni-10.47V (in at.%), which was analyzed using a scanning electron microscope (SEM, JEOL JSM-7001F) with an attached X-ray energy dispersive spectrometer (SEM-EDS). The ingot was fabricated through the vacuum induction melting technique, homogenized at 1473 K for 3 h, and subsequent water quenched. Ingot with a thickness of 7.8 mm was rolled to 0.6 mm at room temperature. The total reduction in thickness after rolling was ~92.3%, and the corresponding true strain is ~2.56. The cold-rolled sample is named as CR. In order to obtain fully recrystallized specimens, the rolled sheets were annealed at temperatures of 1023 K and 1073 K for 20 min, respectively, which were denoted as CR750 and CR800, correspondingly. The flowchart of experimental methods is shown in Figure 1.

Tensile specimens were fabricated from annealed sheets using an electrical discharge machine, with a gauge length of 10 mm, width of 4 mm, and thickness of 0.6 mm. The surfaces of the specimens were further polished to a 2000# grid finish. Tensile tests were conducted at room temperature using a CMT 5105 testing machine, employing an initial strain rate of 10^−3^ s^−1^. Throughout the tensile test, a standard extensometer was employed until the point of fracture. The loading direction is parallel to the rolling direction (RD).

The crystal structure of the samples was analyzed using a Smartlab (Rigaku, Tokyo, Japan) X-ray diffraction (XRD) device, which scanned the angle range of 30°–100° at a rate of 2°/min. The surface of the XRD samples was ground with SiC grit papers, and electropolished in perchlorate alcohol to remove the strained layer in the surface. Microstructural characterizations were performed using a JSM-7001F field emission scanning electron microscope (FE-SEM) equipped with a backscattered electron (BSE) detector. The deformation microstructures after tensile tests were characterized by transmission electron microscope (TEM, JEM-2100F). The TEM samples were twin-jet electropolished using a solution mixture of 10% perchloric acid and 90% ethanol. The electropolishing process was performed at a direct voltage of 20 V and a temperature of 253 K.

## 3. Results

### 3.1. Microstructure of the Annealed Specimens

**Figure 2:**
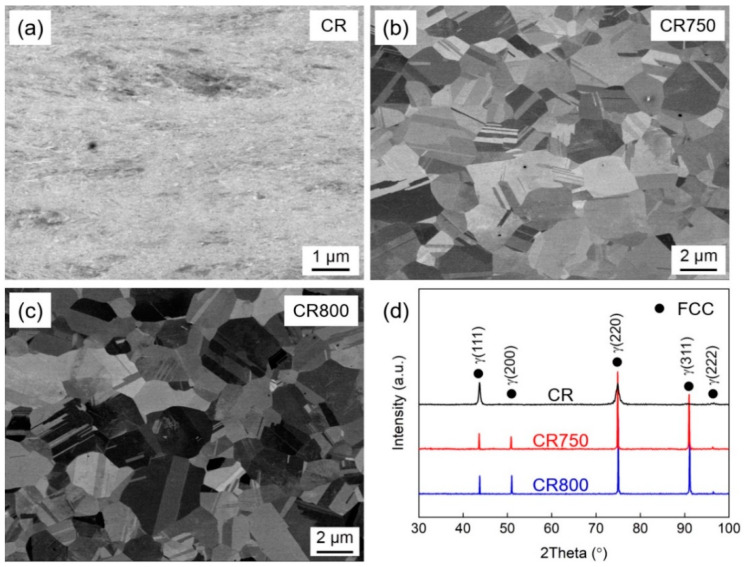
BSE-SEM images and XRD patterns of Co_33_Cr_24_Ni_33_V_10_ alloys. (**a**–**c**) BSE-SEM images of CR, CR750 and CR800, respectively. (**d**) XRD patterns of CR, CR750 and CR800.

Figure 2 illustrates the BSE images and XRD patterns of the CR, CR750, and CR800. CR exhibits typical rolling deformation microstructure characteristics, as shown in Figure 2a. The grains have been severely fragmented after rolling deformation. After annealing treatment, both the CR750 and CR800 have been fully recrystallized, with abundant annealing twins. The average grain sizes of CR750 and CR800 were calculated using the line intercept method, including and excluding twin boundaries (TBs), respectively. The statistical results for CR750 (0.95 ± 0.18 μm, 1.51 ± 0.23 μm) and CR800 (1.08 ± 0.11 μm, 1.78 ± 0.15 μm) are presented. It is noted that CR750 and CR800 shows fully recrystallized FG microstructure, which is quite difficult to achieve due to the rapid grain growth. The XRD patterns in Figure 2d confirm that the three samples obtain FCC single-phase structure. However, MPEAs often tend to form the second phases or intermetallic compounds during heat treatment process. The retained FCC structure in these alloys may be attributed to the insufficient concentration of V, making it difficult to form hexagonal close-packed (HCP) phase after element segregation. These results suggest that the Co_33_Cr_24_Ni_33_V_10_ alloy maintains superior phase stability. The diffraction peak of the alloy rolled only at room temperature is wider than that of the alloy completely recrystallized after annealing, which is due to the greater degree of grain refinement of the alloy after rolling.

### 3.2. Mechanical Properties

**Figure 3:**
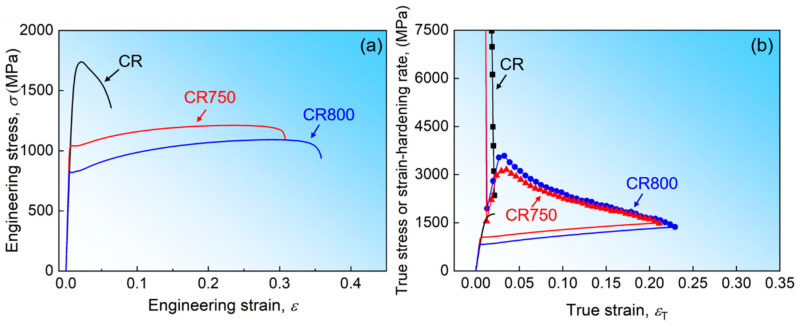
Mechanical properties of the CoCrNiV alloys. (**a**) engineering stress-strain curves; (**b**) strain-hardening curves of CR, CR750 and CR800.

The tensile stress-strain curves and strain-hardening curves of the alloys at room temperature are depicted in Figure 3. The engineering stress-strain curves are depicted in Figure 3a. The CR exhibits a high yield strength of 1513 MPa, but the strain-hardening ability is very limited, and the uniform elongation is only 2.2%. This mechanical response is typical for severely deformed materials. Upon annealing, the CR750 demonstrates a significant increase in uniform elongation, reaching 24.1%. Meanwhile, the yield strength decreases but still remains at 1060 MPa, which is remarkable for single-phase FCC alloys. It is important to note that in the CR800, the relatively large grain size weakens the effect of fine-grain strengthening. This leads to a decrease in yield strength to 846 MPa but an increase in uniform elongation to 29.6%. In the comparison of the mechanical properties among the three states of the alloys, the CR750 exhibits excellent comprehensive mechanical properties. Its uniform elongation is significantly improved compared with the CR, while still maintaining a yield strength above 1 GPa. In contrast, due to the increase of grain size, the ductility of CR800 increases slightly, but the yield strength decreases obviously.

The measurement of the strain-hardening rate is performed using the true stress-strain curve (*Θ* = d*σ*/d*ε*), as shown in Figure 3b. The Considére criterion predicts that necking occurs when *Θ* = *σ*. Therefore, the ductility of the alloy is closely associated with its strain-hardening ability [31]. The CR specimen almost lost its strain-hardening ability after cold rolling deformation. The strain-hardening rate curve of the sample contains only the rapid decline part caused by dynamic recovery in the initial deformation stage. The strain-hardening rate of both the CR750 and CR800 alloys follows a similar pattern, characterized by a multi-stage strain-hardening response. Initially, the strain-hardening rate decreases rapidly, then rises sharply to reach the maximum value. This phenomenon can be attributed to the discontinuous yield behavior of alloys [32]. Subsequently, the strain-hardening rate continues to decrease until necking occurs. Since mobile dislocations have been mostly removed in FG and UFG materials with fully recrystallized structure, the stress required for activating dislocation sources significantly increases. However, the required stress decreases after dislocation multiplication, resulting in a discontinuous yield phenomenon [32].

### 3.3. Microstructure after Tensile Tests

**Figure 4:**
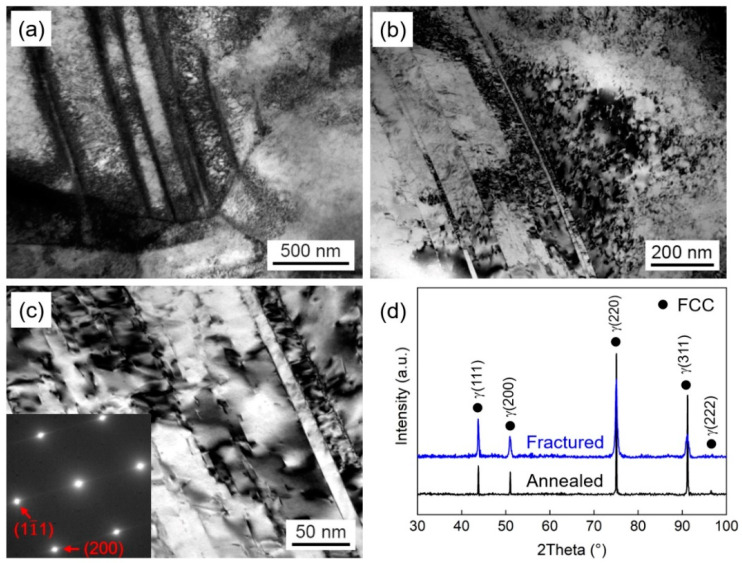
Deformation microstructures of CR750 after tensile tests. (**a**–**c**) TEM images, the SAED pattern of the matrix structure was inserted in (**c**). (**d**) XRD patterns of CR750 alloy before and after deformation.

**Figure 5:**
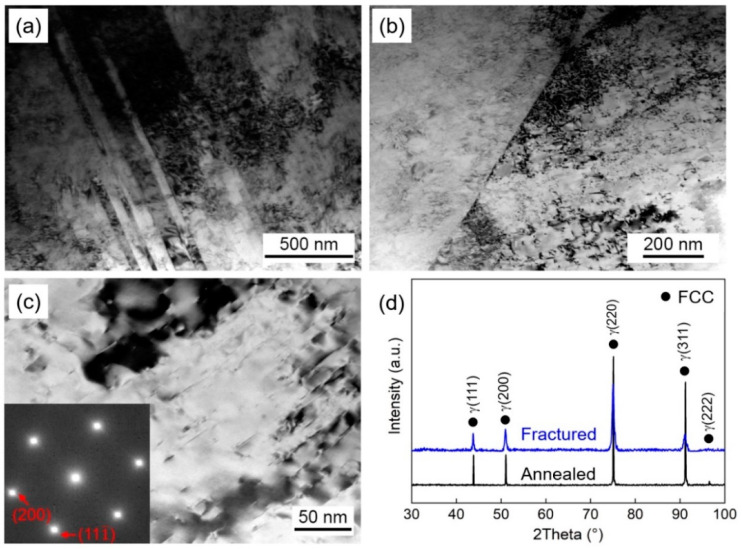
Deformation microstructures of CR800 after tensile tests. (**a**–**c**) TEM images, the SAED pattern of the matrix structure was inserted in (**c**). (**d**) XRD patterns of CR800 alloy before and after deformation.

The formation of defects during the deformation process, such as dislocation entanglement, twins, and stacking faults (SFs), will impede the movement of dislocations, resulting in strain-hardening of alloys [33]. Additionally, SFE influences the mechanical properties of alloys by affecting the deformation mechanisms. In cases where the SFE is low, it can not only generate high-density dislocations but also activate twinning-induced plasticity (TWIP) or transformation-induced plasticity (TRIP) effects [34]. To reveal the reasons for comprehensive mechanical properties of CR750 and CR800, the deformation microstructures after tensile tests were characterized using XRD, TEM, and selected area electron diffraction (SAED). The results are presented in Figure 4 and Figure 5.

The TEM results reveal that both CR750 and CR800 exhibit similar deformation microstructures. Within the grains of both samples, high-density dislocations and SFs were observed, whereas deformation twinning and phase transformation were rarely observed. By examining the XRD patterns in Figure 4a and Figure 5a, it can be clearly observed that the diffraction peaks of the materials after tensile deformation are significantly broadened compared to those of the annealed samples. This can be attributed to the significant presence of dislocations and SFs that are generated during the deformation process. In addition, the tensile deformation may also induce the lattice distortion, which cause the lattice constants in the crystal to be non-uniform, resulting in the broadening of the diffraction peaks. Combining the XRD results with the SAED illustrations in Figure 4d and Figure 5d, it can be inferred that CR750 and CR800 retain FCC single-phase structure after tensile deformation, which confirms the absence of deformation twinning and phase transformation. However, in CoCrNi MPEA with a lower SFE, deformation twinning and phase transformation are often activated during the deformation process. He et al. [35] found that the deformation microstructures were predominantly composed of dislocations and twinning at room temperature. As the temperature decreases to 15 K, twinning and the HCP phase could be clearly distinguished. However, in this study, the absence of twinning or HCP phase can be attributed to the addition of V to increase the SFE of Co_33_Cr_24_Ni_33_V_10_ alloy, which inhibits the TWIP and TRIP effects [30]. The presence of V induces severe lattice distortion. In accordance with classical dislocation theory, significant lattice distortion leads to the deviation of atoms from their equilibrium positions, which affects the nucleation and movement of dislocations [36,37]. When the motion of perfect dislocations is suppressed, it will be dissociated into Shockley partial dislocations, resulting in the formation of SFs. This corresponds to the observed high-density dislocations and SFs in Figure 4 and Figure 5. In the plastic deformation process of lower SFE alloys, the perfect dislocation is prone to be dissociated into partial dislocations, leading to the formation of numerous SFs. The interaction between SFs and dislocations hinders dislocation movement and reduces the free path of dislocations [38], which improves the strain-hardening rate of the alloys.

## 4. Discussion

### 4.1. Formation of FCC Single-Phase FG Structure

Chen et al. [30] proposed a new low SFE V_0.5_Cr_0.5_CoNi alloy, and observed that an increased propensity for high-density SFs within the FCC matrix after cold rolling. During the annealing process, solutes with a greater negative mixing enthalpy (specifically, the V element) readily segregated into SFs via the Suzuki mechanism. As a result, the formation of the HCP phase was promoted at an intermediate annealing temperature [39]. Salishchev et al. [40] introduced a certain content of V element into CoCrFeMnNi and CoCrFeNi alloys. Since V is a stronger σ phase-forming element compared to Cr, the addition of V improved the instability of the FCC phase [41]. Consequently, it becomes easier for the alloys to form a V-rich σ phase, with an increase in the V content, there is a corresponding increase in the volume fraction of the σ phase. The results showed that when a certain amount V was added to the CoCrNi alloys, the annealed alloys exhibited V segregation behavior and the formation of the second phase [30]. In contrast, when the V content was reduced to 10% in this study, the locally enriched element concentration was insufficient to form the second phase, thus resulting in the retention of a single-phase FCC structure after annealing.

According to experimental results and theoretical calculations, it has been determined that the influence of Cr on reducing the SFE of the alloys was greater than V [30]. Compared to the equiatomic CoCrNi MPEA, the Co_33_Cr_24_Ni_33_V_10_ alloy exhibits a higher SFE, the deformed structure of the alloy after cold rolling exhibits high-density dislocations and SFs, without activating twinning or phase transformation. Consequently, the alloy maintains its single-phase FCC structure even after deformation and retains excellent mechanical properties. Furthermore, the development of the FG microstructure in the CR750 and CR800 can be attributed to the characteristic hysteresis diffusion effect observed in HEAs and MEAs.

### 4.2. Deformation Mechanisms

The CR750 alloy exhibits outstanding comprehensive mechanical properties, with yield strength up to 1060 MPa and remarkable uniform elongation of 24.1% at room temperature. Tensile deformation results in the formation of microstructures primarily composed of highly entangled dislocations and SFs. However, there is no formation of deformation twinning or HCP phase. Based on these observations, the deformation behavior and exceptional ductility of the CR750 can be elucidated.

Firstly, the SFE plays a crucial role in dictating the deformation mechanisms observed in metals and alloys during plastic deformation. A higher SFE results in a narrow SF width and frequent cross-slip, leading to a dominant wavy slip behavior. Conversely, When the SFE of the alloys is lower, the partial dislocations with wide spacing will inhibit the cross-slip, and the dislocation slip shows a planar slip mode, and even the TWIP and TRIP effects appear, which improves the comprehensive mechanical properties of the alloys [34]. In CoCrNi alloy with a low SFE, a large number of SFs are often generated during the deformation process, leading to deformation twinning [16]. Under specific temperature and strain rate conditions, deformation-induced martensite transformation may also occur [34]. However, theoretical calculations indicated that the SFE of VCoNi was higher than that of CoCrNi when the Cr was replaced with the V [30]. It has been confirmed that Cr has a greater effect in reducing the SFE compared to V. In this study, as for Co_33_Cr_24_Ni_33_V_10_ alloy, the deformed microstructures do not contain twinning and HCP phase after tensile tests. Shockley partial dislocations are more likely to dissociate from full dislocations, forming SFs during the plastic deformation process. The reason is that when Cr is replaced by V in the Co_33_Cr_24_Ni_33_V_10_ alloy, it causes an increase in the SFE compared to the equiatomic CoCrNi alloy. As a result, the Co_33_Cr_24_Ni_33_V_10_ alloy does not exhibit deformation-induced twinning phenomenon during tensile process at room temperature. The deformation mechanisms are primarily governed by dislocations and SFs. Meanwhile, the twinning and HCP phase are formed by the initial SFs as the nucleation sites, and partial dislocations slip through different pathways [35]. Consequently, an increased SFE makes it challenging for twinning and HCP phase to form, leading to the presence of high-density dislocation tangles and SFs after deformation. Secondly, there exists a relationship between the grain size and the critical stress necessary for twinning. The larger the grain size, the smaller the critical stress required for twinning formation [37]. Therefore, the FG structure of the Co_33_Cr_24_Ni_33_V_10_ alloy makes twinning difficult.

Although the addition of V increases the SFE of the Co_33_Cr_24_Ni_33_V_10_ alloy, but it still maintains a low SFE level, which is favorable for the activation of SFs during the plastic deformation process. Besides dislocations interaction, SFs can also contribute to continuous strain-hardening [42]. SFs interact with dislocations and effectively impede dislocations motion. When dislocations are intercepted by SFs, the SFs may either decompose into smaller fragmented sections or absorb the impeded dislocations and generate new dislocations [43]. These new dislocations can penetrate SFs or cross-slip along them, but both mechanisms require higher stress levels [44]. Therefore, the fully recrystallized FG structure, dislocation slip, and the interaction between SFs and dislocations in the CR750 alloy collectively enhance the strain-hardening ability and plastic deformation capacity. Therefore, they endow the alloy with excellent strength and ductility.

### 4.3. Strengthening Mechanisms

The CR750 alloy, with its single-phase FCC structure, demonstrates excellent mechanical properties, including an outstanding yield strength of up to 1060 MPa at room temperature. The ultra-high yield strength can be mainly attributed to two factors: the FG structure of the alloy and the solid solution strengthening effect introduced by the V element. According to the Hall-Petch relationship, when the grain size of the alloys is smaller, a larger number of grain boundaries inhibit dislocation motion, thus enhancing the strength of the alloys. Previous research has indicated that refining the grain size to 503 nm could result in a yield strength of 888 MPa for the fully recrystallized CoCrFeMnNi alloy [45]. However, even with a finer grain size, the yield strength of the CoCrFeMnNi alloy is considerably inferior to that of the CR750 alloy. This implies that, apart from grain size refinement, the enhancement of the yield strength in the CR750 alloy is significantly influenced by the presence of additional strengthening mechanisms.

**Figure 6:**
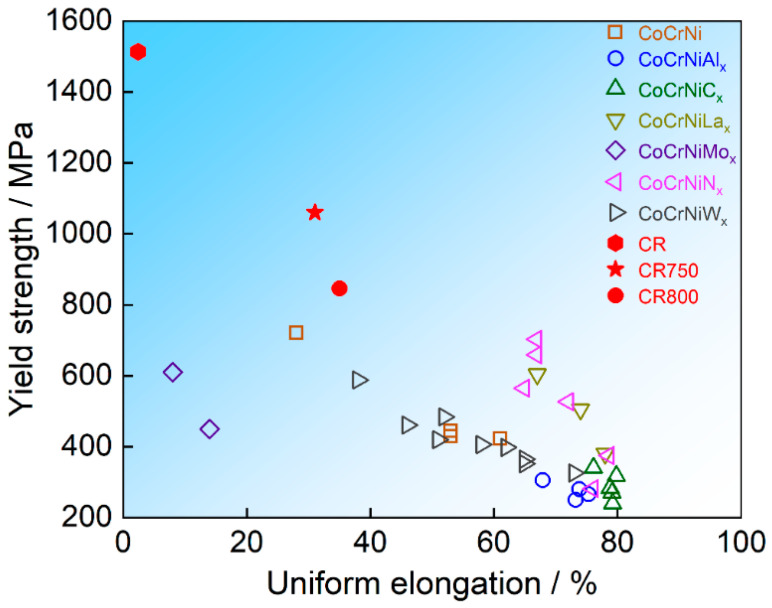
Mechanical properties of CoCrNi MPEAs with the fourth elements [41,46,47,48,49,50,51].

MPEAs exhibit stronger lattice distortion compared to traditional materials, which results in a higher resistance for dislocation movement. This phenomenon highlights the significant solid solution strengthening effect of MPEAs. Based on the theory of dislocation and interaction energy in disordered alloys, the large difference in atomic size between V and other main elements enables it to exert a solid solution strengthening effect, thereby enhancing the mechanical properties of FCC HEAs [28]. The presence of V in FCC alloys leads to variations in atomic spacing, inducing localized fluctuations in Peierls’ potential for dislocation glide and consequently increasing frictional stress [18,29]. Calculation predictions indicate that the addition of V to the CoCrFeMnNi alloys is capable of producing large atomic volume misfits, and thus, V can produce higher strength than other alloy elements [38]. Figure 6 illustrates a comparison of the mechanical properties of the CoCrNi alloys after incorporating the fourth elements. But in contrast, the Co_33_Cr_24_Ni_33_V_10_ alloy studied here demonstrates exceptional comprehensive mechanical properties. In summary, the ultra-high yield strength of CR750 is attributed to the combined strengthening effects of FG structure and high lattice friction stress.

## 5. Conclusions

Fully recrystallized FG Co_33_Cr_24_Ni_33_V_10_ alloys, CR750 and CR800, were produced by cold rolling at room temperature and heat treatment. By conducting tensile tests and microstructure characterization, the main conclusions can be summarized as follows:

1. The Co_33_Cr_24_Ni_33_V_10_ alloy maintained a single-phase FCC structure and exhibited superior phase stability throughout the processes of rolling, heat treatment, and tensile deformation.
2. The primary deformation mechanisms of the CR750 and CR800 alloys were dislocation slip and SFs, with no occurrence of twinning or phase transformation.
3. The fully recrystallized FG structure of CR750 alloy with the minimum mean grain size of 0.95 μm exhibited a high yield strength of 1060 MPa and a uniform elongation of 24.1%. The exceptional strength can be attributed to the presence of the FG structure and solid solution strengthening effect, while the remarkable ductility arises from the excellent strain-hardening ability primarily governed by dislocations and SFs.

## Data Availability

The data presented in this study are available on request from the corresponding author.

## References

[1] Yeh J.W., Chen S.K., Lin S.J., Gan J.Y., Chin T.S., Shun T.T., Tsau C.H., Chang S.Y. (2004). Nanostructured High-Entropy Alloys with Multiple Principal Elements: Novel Alloy Design Concepts and Outcomes. Adv. Eng. Mater..

[2] Fu A., Liu B., Lu W.J., Liu B., Li J., Fang Q.H., Li Z.M., Liu Y. (2020). A novel supersaturated medium entropy alloy with superior tensile properties and corrosion resistance. Scr. Mater..

[3] Xu H.Q., Li Z.Y., Zhou W., Ma L.H., Zhang M.D., Li G. (2021). Aluminum and titanium alloyed non-equiatomic Co–Fe–Ni medium-entropy alloy with ultra high strength and hardness. Mater. Sci. Eng. A.

[4] Feng X.B., Yang H.K., Fan R., Zhang W.Q., Meng F.L., Gan B., Lu Y. (2020). Heavily twinned CoCrNi medium-entropy alloy with superior strength and crack resistance. Mater. Sci. Eng. A.

[5] Wang J.Y., Yang H.L., Huang H., Zou J.P., Ji S.X., Liu Z.L. (2020). High strength-ductility Co_23_Cr_23_Ni_23_Mn_31_ medium-entropy alloy achieved via defect engineering. Mater. Sci. Eng. A.

[6] Wang Y., Yang H., Zhang Z., Meng X., Cheng T., Qin G., Li S. (2023). Far-from-equilibrium electrosynthesis ramifies high-entropy alloy for alkaline hydrogen evolution. J. Mater. Sci. Technol..

[7] Liu J.P., Chen J.X., Liu T.W., Li C., Chen Y., Dai L.H. (2020). Superior strength-ductility CoCrNi medium-entropy alloy wire. Scr. Mater..

[8] Hu M.Y., Ouyang X.M., Yin F.C., Zhao X., Zhang Z.C., Wang X.M. (2023). Effect of Boronizing on the Microstructure and Mechanical Properties of CoCrFeNiMn High-Entropy Alloy. Materials.

[9] Wang M.L., Lu Y.P., Wang T.M., Zhang C., Cao Z.Q., Li T.J., Liaw P.K. (2021). A novel bulk eutectic high-entropy alloy with outstanding as-cast specific yield strengths at elevated temperatures. Scr. Mater..

[10] Lu Y.P., Gao X.Z., Jiang L., Chen Z.M., Wang T.M., Jie J.C., Kang H.J., Zhang Y.B., Guo S., Ruan H.H. (2017). Directly cast bulk eutectic and near-eutectic high entropy alloys with balanced strength and ductility in a wide temperature range. Acta Mater..

[11] Gorr B., Schellert S., Müller F., Christ H.J., Kauffmann A., Heilmaier M. (2021). Current Status of Research on the Oxidation Behavior of Refractory High Entropy Alloys. Adv. Eng. Mater..

[12] Anne B.R., Shaik S., Tanaka M., Basu A. (2021). A crucial review on recent updates of oxidation behavior in high entropy alloys. SN Appl. Sci..

[13] Peng Z., Fan Z.Z., Abdullah M.R., Ren C.C., Li J.F., Gong P. (2023). Corrosion Resistance Enhancement of CoCrFeMnNi High-Entropy Alloy with WC Particle Reinforcements via Laser Melting Deposition. Materials.

[14] Wang Y., Li D., Yang J.S., Jin J.S., Zhang M., Wang X.Y., Li B., Hu Z.G., Gong P. (2023). Effect of Grain Size on the Tribological Behavior of CoCrFeMnNi High Entropy Alloy. Materials.

[15] Lu Y.P., Huang H.F., Gao X.Z., Ren G.L., Gao J., Zhang H.Z., Zheng S.J., Jin Q.Q., Zhao Y.H., Lu C.Y. (2019). A promising new class of irradiation tolerant materials: Ti_2_ZrHfV_0.5_Mo_0.2_ high-entropy alloy. J. Mater. Sci. Technol..

[16] Otto F., Dlouhý A., Somsen C., Bei H., Eggeler G., George E.P. (2013). The influences of temperature and microstructure on the tensile properties of a CoCrFeMnNi high-entropy alloy. Acta Mater..

[17] Laplanche G., Kostka A., Reinhart C., Hunfeld J., Eggeler G., George E.P. (2017). Reasons for the superior mechanical properties of medium-entropy CrCoNi compared to high-entropy CrMnFeCoNi. Acta Mater..

[18] Li Z.T., Ma S.H., Zhao S.J., Zhang W.D., Peng F., Li Q., Yang T., Wu C.-Y., Wei D.X., Chou Y.-C. (2023). Achieving superb strength in single-phase FCC alloys via maximizing volume misfit. Mater. Today.

[19] Shen R., Ni Z., Peng S., Yan H., Tian Y. (2023). Effects of V Addition on the Deformation Mechanism and Mechanical Properties of Non-Equiatomic CoCrNi Medium-Entropy Alloys. Materials.

[20] Mustafa F., Egilmez M., Abuzaid W., El-Khatib S., Nawaz T., Ahmad S., Alagoz S. (2023). Strange Metallicity and Magnetic Order in the CoNi (Cr/V) Medium-Entropy Alloy System. Materials.

[21] Moravcik I., Hornik V., Minárik P., Li L.L., Dlouhy I., Janovska M., Raabe D., Li Z.M. (2020). Interstitial doping enhances the strength-ductility synergy in a CoCrNi medium entropy alloy. Mater. Sci. Eng. A.

[22] Li Z., Long X., Zhang S.Y., Deng H.W., Zhang M., Zhang T. (2023). Ultrasonic provoked network-like intragrain carbide precipitations strengthen and ductilize a C-doped CoCrNi medium entropy alloy. Scr. Mater..

[23] Huang X.L., Huang L.P., Peng H.L., Liu Y., Liu B., Li S. (2021). Enhancing strength-ductility synergy in a casting non-equiatomic NiCoCr-based high-entropy alloy by Al and Ti combination addition. Scr. Mater..

[24] Zheng M.Y., Li C.W., Ye Z.H., Zhang X.Y., Yang X.D., Wang Q., Gu J.F. (2023). Strength-ductility synergy of additively manufactured (CoCrNi)_87_Al_13_ medium entropy alloy with heterogeneous multiphase microstructure. Scr. Mater..

[25] Miao J.W., Li T.X., Li Q., Chen X.H., Ren Z., Lu Y.P. (2023). Enhanced Surface Properties of the Al_0.65_CoCrFeNi High-Entropy Alloy via Laser Remelting. Materials.

[26] Tian S., Liu Z.B., Fu R.L., Dong C.F., Wang X.H. (2022). Effect of Organizational Evolution on the Stress Corrosion Cracking of the Cr-Co-Ni-Mo Series of Ultra-High Strength Stainless Steel. Materials.

[27] He J.Y., Wu X.X., Guo Y.L., Makineni S.K. (2021). On the compositional and structural redistribution during partial recrystallisation: A case of σ-phase precipitation in a Mo-doped NiCoCr medium-entropy alloy. Scr. Mater..

[28] Yin B.L., Maresca F., Curtin W.A. (2020). Vanadium is an optimal element for strengthening in both fcc and bcc high-entropy alloys. Acta Mater..

[29] Sohn S.S., Kwiatkowski da Silva A., Ikeda Y., Körmann F., Lu W., Choi W.S., Gault B., Ponge D., Neugebauer J., Raabe D. (2019). Ultrastrong Medium-Entropy Single-Phase Alloys Designed via Severe Lattice Distortion. Adv. Mater..

[30] Chen Z., Xie H.B., Yan H.L., Pang X.Y., Wang Y.H., Wu G.L., Zhang L.J., Tang H., Gao B., Yang B. (2022). Towards ultrastrong and ductile medium-entropy alloy through dual-phase ultrafine-grained architecture. J. Mater. Sci. Technol..

[31] Shao C.W., Zhang P., Zhu Y.K., Zhang Z.J., Tian Y.Z., Zhang Z.F. (2018). Simultaneous improvement of strength and plasticity: Additional work-hardening from gradient microstructure. Acta Mater..

[32] Tian Y.Z., Gao S., Zhao L.J., Lu S., Pippan R., Zhang Z.F., Tsuji N. (2018). Remarkable transitions of yield behavior and Lüders deformation in pure Cu by changing grain sizes. Scr. Mater..

[33] Sun S.J., Tian Y.Z., Zhang Z.F. (2022). Strengthening and Toughening Mechanisms of Precipitation- Hardened Fe_53_Mn_15_Ni_15_Cr_10_Al_4_Ti_2_C_1_ High-Entropy Alloy. Acta Metall. Sin..

[34] Yi H.L., Xie R.Y., Zhang Y.F., Wang L.Q., Tan M., Li T., Wei D.X. (2022). Tuning Microstructure and Mechanical Performance of a Co-Rich Transformation-Induced Plasticity High Entropy Alloy. Materials.

[35] He H.Y., Naeem M., Zhang F., Zhao Y.L., Harjo S., Kawasaki T., Wang B., Wu X., Lan S., Wu Z. (2021). Stacking Fault Driven Phase Transformation in CrCoNi Medium Entropy Alloy. Nano Lett..

[36] Gong D., Cao Y.F., Deng X.B., Jiang L.T. (2022). Higher proportional limit of SiC/Al composites with nano-scaled stacking faults. Compos. Commun..

[37] An X.L., Ni S., Song M., Liao X.Z. (2020). Deformation Twinning and detwinning in face-centred cubic metallic materials. Adv. Eng. Mater..

[38] Jo Y.H., Jung S., Choi W.M., Sohn S.S., Kim H.S., Lee B.J., Kim N.J., Lee S. (2017). Cryogenic strength improvement by utilizing room-temperature deformation twinning in a partially recrystallized VCrMnFeCoNi high-entropy alloy. Nat. Commun..

[39] Jo Y.H., Choi W.M., Kim D.G., Zargaran A., Sohn S.S., Kim H.S., Lee B.J., Kim N.J., Lee S. (2019). FCC to BCC transformation-induced plasticity based on thermodynamic phase stability in novel V_10_Cr_10_Fe_45_Co_x_Ni_35-x_ medium-entropy alloys. Sci. Rep..

[40] Salishchev G.A., Tikhonovsky M.A., Shaysultanov D.G., Stepanov N.D., Kuznetsov A.V., Kolodiy I.V., Tortika A.S., Senkov O.N. (2014). Effect of Mn and V on structure and mechanical properties of high-entropy alloys based on CoCrFeNi system. J. Alloys Compd..

[41] Stepanov N.D., Shaysultanov D.G., Salishchev G.A., Tikhonovsky M.A., Oleynik E.E., Tortika A.S., Senkov O.N. (2015). Effect of V content on microstructure and mechanical properties of the CoCrFeMnNiV_x_ high entropy alloys. J. Alloys Compd..

[42] Lee C.F., Shun T.T. (2021). Effects of the Replacement of Co with Ni on the Microstructure, Mechanical Properties, and Age Hardening of AlCo_1−x_CrFeNi_1+x_ High-Entropy Alloys. Materials.

[43] Su R.Z., Neffati D.J., Zhang Y.F., Cho J.H., Li J., Wang H.Y., Kulkarni Y., Zhang X.H. (2021). The influence of stacking faults on mechanical behavior of advanced materials. Mater. Sci. Eng. A.

[44] Guo B.S., Song M., Zhang X.M., Liu Y.Z., Cen X., Chen B., Li W. (2021). Exploiting the synergic strengthening effects of stacking faults in carbon nanotubes reinforced aluminum matrix composites for enhanced mechanical properties. Compos. B.

[45] Sun S.J., Tian Y.Z., Lin H.R., Dong X.G., Wang Y.H., Zhang Z.J., Zhang Z.F. (2017). Enhanced strength and ductility of bulk CoCrFeMnNi high entropy alloy having fully recrystallized ultrafine-grained structure. Mater. Des..

[46] Agustianingrum M.P., Yoshida S., Tsuji N., Park N. (2019). Effect of aluminum addition on solid solution strengthening in CoCrNi medium-entropy alloy. J. Alloys Compd..

[47] Shang Y.Y., Wu Y., He J.Y., Zhu X.Y., Liu S.F., Huang H.L., An K., Chen Y., Jiang S.H., Wang H. (2019). Solving the strength-ductility tradeoff in the medium-entropy NiCoCr alloy via interstitial strengthening of carbon. Intermetallics.

[48] Chan S.N., Hsueh C.-H. (2022). Effects of La addition on the microstructure and mechanical properties of CoCrNi medium entropy alloy. J. Alloys Compd..

[49] Li N., Chen W.T., He J.Y., Gu J., Wang Z.W., Li Y., Song M. (2021). Dynamic deformation behavior and microstructure evolution of CoCrNiMo_x_ medium entropy alloys. Mater. Sci. Eng. A.

[50] Moravcik I., Hadraba H., Li L.L., Dlouhy I., Raabe D., Li Z.M. (2020). Yield strength increase of a CoCrNi medium entropy alloy by interstitial nitrogen doping at maintained ductility. Scr. Mater..

[51] Chang R.B., Fang W., Bai X., Xia C.Q., Zhang X., Yu H.Y., Liu B.X., Yin F.X. (2019). Effects of tungsten additions on the microstructure and mechanical properties of CoCrNi medium entropy alloys. J. Alloys Compd..

